# La Crosse Virus Circulation in Virginia, Assessed via Serosurveillance in Wildlife Species

**DOI:** 10.3390/idr15040036

**Published:** 2023-06-30

**Authors:** Lindsey R. Faw, Jennifer Riley, Gillian Eastwood

**Affiliations:** 1 Department Entomology, Virginia Polytechnic Institute and State University, Blacksburg, VA 24061, USA; 2 Center for Emerging, Zoonotic, and Arthropod-borne Pathogens (CeZAP), Virginia Tech, Blacksburg, VA 24060, USA; 3 Blue Ridge Wildlife Center, Boyce, VA 22620, USA; 4 The Global Change Center at Virginia Tech, Blacksburg, VA 24061, USA

**Keywords:** La Crosse virus, serology, arbovirus, Virginia, vector-borne disease, mosquito, wildlife

## Abstract

Mosquito-borne La Crosse virus (LACV; family: *Peribunyaviridae*) is the leading cause of pediatric arboviral encephalitis in the United States, with clinical cases generally centered in the Midwest and Appalachian regions. Incidence of LACV cases in Appalachian states has increased, such that the region currently represents the majority of reported LACV cases in the USA. The amount of reported LACV cases from Virginia, however, is minimal compared to neighboring states such as North Carolina, West Virginia, and Tennessee, and non-Appalachian regions of Virginia are understudied. Here we examine the hypothesis that LACV is circulating widely in Virginia, despite a low clinical case report rate, and that the virus is circulating in areas not associated with LACV disease. In this study, we screened local mammalian wildlife in northwestern counties of Virginia using passive surveillance via patients submitted to wildlife rehabilitation centers. Blood sera (527 samples; 9 species, 8 genera) collected between October 2019 and December 2022 were screened for neutralizing antibodies against LACV, indicating prior exposure to the virus. We found an overall LACV seroprevalence of 1.90% among all wild mammals examined and reveal evidence of LACV exposure in several wild species not generally associated with LACV, including eastern cottontails and red foxes, along with established reservoirs, eastern gray squirrels, although there was no serological evidence in chipmunks. These data indicate the circulation of LACV in Virginia outside of Appalachian counties, however, at a lower rate than reported for endemic areas within the state and in other states.

## 1. Introduction

Mosquito-borne diseases are of public health concern worldwide [1,2]. Of the etiological agents of these diseases, arboviruses (viruses transmitted by arthropods) are a major contributor to human illness and are surpassing *Plasmodium* spp. (malaria) as the most common mosquito-borne illness globally [3]. In the United States (USA) infectious diseases including arboviruses increase or emerge as a response to anthropogenic, ecological, and environmental factors [2,4]. One notable arboviral agent, La Crosse orthobunyavirus (LACV), is the leading cause of pediatric arboviral encephalitis in the USA [5]. LACV is a tripartite, negative-sense RNA virus in the California serogroup of the genus *Orthobunyavirus* (family: *Peribunyaviridae*) [6]. 

LACV was first isolated from the brain of a child who died from encephalitis in 1960 in La Crosse, Wisconsin, from where the virus name was derived [7]. Now known as the causative agent of La Crosse encephalitis (LACE), LACV infection is a reportable disease, predominately occurring in the Midwest and Appalachian regions, averaging between 31 and 84 cases annually [8]. Clinical manifestations include headache, fever, vomiting, seizures, disorientation, swelling of the brain and meninges, and other neurological symptoms that primarily affect children under 16 years of age [9,10,11,12]. LACV can also cause a non-encephalitic clinical infection, likely underreported, which is characterized by fever and headache, or often completely asymptomatic [9,12]. 

LACV is understood to be maintained in a sylvatic cycle between *Aedes* spp. mosquito vectors, the native primary vector being *Aedes triseriatus*, and vertebrate species of the family *Sciuridae* acting as reservoir hosts (e.g., chipmunks, squirrels, and groundhogs) [13]. People do not develop high enough viremias for onward transmission of the virus; thus, humans are considered ‘dead-end’ hosts. There are three viral lineages of LACV, with lineage I strains circulating in the Appalachian region of the USA. In Appalachia, an area in which *Ae. triseriatus* is widespread, LACV has been identified as an emerging threat [14,15,16,17]; it is suggested that the increase in both LACV prevalence in mosquitoes and LACE cases in the Appalachian region may be related to anthropogenic changes in the environment, such as climate change and land use, that facilitate the invasion of invasive mosquitoes and their interaction with humans [15,16,17,18,19]. Furthermore, although *Ae. triseriatus* is the native LACV vector, field isolations of LACV have been made from the invasive mosquito species, *Aedes albopictus* and *Aedes japonicus*, and both species have been demonstrated to be competent vectors via laboratory studies; the role of additional vector species is suggested for the increased LACE cases in the Appalachian region [16,20,21,22,23]. 

Around 80% of neuroinvasive LACV (i.e., LACE) cases reported to the United States Centers for Disease Control and Prevention (CDC) hail from Appalachia; Appalachia now outnumbers the Midwest in terms of incidence of LACV infections [16,18,24]. Counties along the southwestern and western edge of Virginia fall within Appalachia. The neighboring states of North Carolina (NC), Tennessee (TN), and West Virginia (WV) reported 179, 117, and 68 LACV cases to the CDC between 2011 and 2020, respectively [8]. However, for Virginia (VA), only nine LACV cases were reported to the CDC in the same period [8]. Areas outside of the Appalachian region in VA are understudied for arboviruses due to a perceived lack of risk in comparison to other high-risk areas such as those of deeper Appalachia. However, there were cases of LACV (both neuroinvasive and other) reported from Fairfax County in 2005 and 2013 [25], indicating that LACV activity occurs also in Northern VA or, alternatively, that travel cases imported from other regions occur. To verify local circulation and assess the risks in this region further, we sought to identify the presence of LACV in the northern and northwestern part of the state by considering seroprevalence in wild mammals. 

Serosurveillance is a useful tool to determine the geographical circulation of a virus, as well as assess exposure of vertebrate hosts to a pathogen; the methodology has been utilized with arboviruses such as Eastern equine encephalitis virus [26,27], chikungunya [28], and West Nile virus [27,29], among others. Serosurveillance can provide information about past and current circulation of specific viruses within an area through the detection of neutralizing antibodies (Nab) against the virus in blood serum. Examining seroprevalence rates against LACV in local vertebrate species in Virginia aimed to further elucidate the geographical distribution of this arbovirus. Although data on LACV seroprevalence is sparse, there is a suggestion that seroprevalence of LACV in host species, along with that of Jamestown Canyon virus (JCV), another California serogroup virus, mirrors that of human cases [30,31]. If this is true for LACV, serosurveillance of mammalian hosts will be a good proxy for human illness. Here, we thus examine LACV exposure in local wildlife populations of Northern and Northwestern Virginia, to improve understanding of the circulation of this arbovirus in the region and gain insight on the potential risks for human exposure to the virus.

## 2. Materials and Methods

### 2.1. Sample Collection

Blood was collected from small- and meso-mammals between October 2019 and December 2022. Individuals were patients at three wildlife rehabilitation centers in VA (predominantly submissions to Blue Ridge Wildlife Center, Boyce, VA, USA), road-kill casualties, or sampled via local wildlife control (approvals via VT IACUC (protocol code #20-026) and VADGIF Permit (# 069872) were in place.

A total of 527 samples were transferred to the Virginia Tech Vector-borne Disease Ecology Laboratory for serological analysis. Sera was separated from blood using microtainer serum-separating collection vials (BD, Franklin Lakes, NJ, USA), and aliquots of sera were heat-inactivated at 56 °C for 45 min to remove any active proteins that may interfere with antibody testing, then diluted 1:10 in Dulbecco’s modified essential medium (DMEM) cell culture medium supplemented with 2% fetal bovine serum (FBS) and 1% 100X Penicillin/Streptomycin, here forward, 2% DMEM.

### 2.2. Power Analysis

A power analysis was performed following the following formula [32], in which 𝛃 is the confidence in detecting a LACV-positive sample in the population, d/N is the number of diseased individuals in the population when N is greater than 1000, and n is the sample size needed. All d/N values were calculated using relevant calculations from previous literature [33,34,35,36,37].

(1) $$n=\frac{log\left(1-\beta \right)}{log\left(1-\left(\frac{d}{N}\right)\right)}$$

Based on this power analysis, the following minimum sample sizes needed to be acquired to adequately detect positive samples: 50 chipmunks at a 5.9% seroprevalence rate [33], 50 eastern gray squirrels at a 5.9% seroprevalence [33], 50 flying squirrels at a 5.9% seroprevalence rate [33], and 29 cottontails at a 10% seroprevalence rate [38]. There are no reported rates for wild red foxes or groundhogs, therefore the seroprevalence rate of 5.9% as listed for other mammals was used to calculate power analysis; 50 red foxes and 50 groundhogs were needed for a 95% confidence estimate of exposure.

### 2.3. Serological Analysis

Serum samples were tested for the presence of neutralizing antibodies against LACV using plaque reduction neutralization tests (PRNTs). Briefly, diluted serum was challenged 1:1 with LACV (strain 78V-13193, previously passaged once in suckling mouse, twice in Vero cells and stock titer determined) diluted to 800 plaque-forming units (pfu)/mL and incubated at 37 °C with 5% CO_2_ for one hour. Following the challenge period, confluent Vero-76 cells (ATCC, Manassas, VA, USA), seeded in 12-well culture plates, were inoculated with 50 uL of each virus-sera mix, incubated, and rocked every 10 min for one hour, before a solid overlay was applied consisting of 1:5 (0.4%) SeaKem^®^ agarose (Lonza, Basal, Switzerland) in 2% DMEM. Negative controls consisted of both no inoculate and 2% DMEM inoculate. Positive controls consisted of a 2-fold serial dilution of LACV 78V-13193 (starting at 800 pfu) diluted until 1:64, as well as a 1:40 and 1:80 dilution of LACV rabbit antisera (kindly provided by CDC, Atlanta, GA, USA). After three days further incubation at 37 °C, plates were fixed with 10% formaldehyde, then stained with 0.1% crystal violet in 10% phosphate-buffered formalin. Seropositive samples were determined using an 80% plaque reduction threshold, PRNT(80), at a minimum serum titer of 1:40. Exact end-point titers of these samples were then established by PRNT using serial 2-fold dilutions of the sera.

## 3. Results

Of 527 serum samples collected and tested for evidence of LACV exposure, wildlife species composition comprised 264 *Sciurus carolinensis* (eastern gray squirrel), 131 *Sylvilagus floridanus* (eastern cottontail), 56 *Marmota monax* (groundhog), 39 *Vulpes vulpes fulva* (red fox), 23 *Tamias striatus* (eastern chipmunk), 5 *Sciurus niger* (eastern fox squirrel), 5 *Glaucomys volans* (Southern flying squirrel), 3 *Castor canadensis* (American beaver), and 1 *Urocyon cinereoargenteus* (gray fox) (Table 1). 

**Figure 1:**
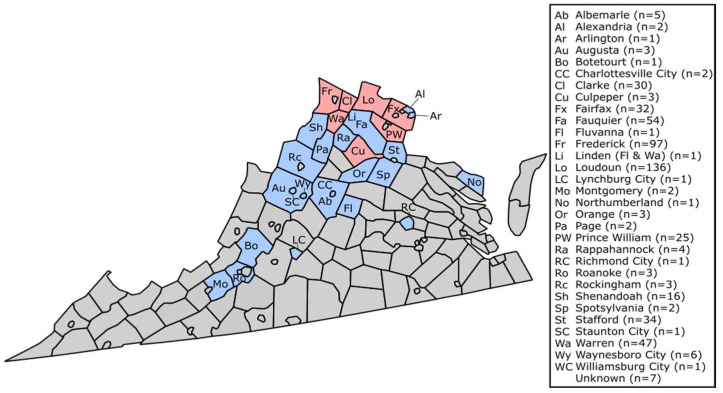
Geographic distribution of study samples. Red counties represent counties yielding LACV-seropositive individuals. Blue counties represent counties where samples originated but where no evidence of LACV was determined.

Ten individuals were LACV-seropositive at an 80% plaque reduction at a serum titer of 1:40 or greater. Details of those specimens are listed in Table 2, with an overall seroprevalence rate of 1.90% across all wild mammals tested. Eastern gray squirrels (*n* = 264 tested) showed a LACV-seroprevalence of 1.89%, eastern cottontails (*n* = 131) had a 2.29% seroprevalence, groundhogs (*n* = 56) had a 1.79% seroprevalence, and red foxes (*n* = 39) had a seroprevalence of 2.56%; none of 23 chipmunks screened showed neutralizing antibodies against LACV (Table 1). Figure 1 shows the geographical location of 24 counties and 7 independent cities in VA from where the wildlife samples were acquired; four samples came from unknown locations. LACV-seropositive samples were detected from Loudon (*n* = 5), Frederick (*n* = 1), Prince William (*n* = 1), Warren (*n* = 1), Fairfax (*n* = 1), and Culpeper (*n* = 1) Counties; (Figure 1, Table 2).

## 4. Discussion

This study explored the prevalence of LACV in local wildlife to evidence the circulation of this mosquito-borne virus in VA. Our data indicate that multiple species in Northern Virginia have been exposed to LACV infection, with an overall LACV seroprevalence of 1.90% across four of nine different mammal species assessed.

We reveal exposure to LACV in four mammalian species (eastern gray squirrel, eastern cottontail, groundhog, and red fox). Other mammalian wildlife species tested here (eastern fox squirrel, American beaver, eastern flying squirrel, eastern chipmunk, and gray fox) did not show evidence of LACV exposure; these seronegative species may have lacked adequate sample counts to demonstrate true exposures, or, alternatively, they are less likely to interact with LACV-infected mosquitoes. Additional studies could elude the exposure of these species further. 

An assumed reservoir host for LACV maintenance and transmission is the eastern chipmunk (Order: Rodentia, Family: *Sciuridae*) [37]. Thus, it is surprising that we did not detect any seropositive individuals, since we have shown that the virus is circulating in the region via other species of wildlife. Our finding further contrasts with a 2015 LACV study conducted in Montgomery County, VA, at the southern tip of our study area, where seroprevalence rates of 13% (*n* = 38) were reported for chipmunks [32]. Our lower sample size of 23 may have been inadequate (50 being aimed for by power analysis) to detect LACV-exposed individuals, and our current study did not include any chipmunk samples from Southwest VA. The lack of seropositivity detected in individuals here may indicate that chipmunks of Northern VA are not exposed, are playing a lesser role in the transmission of LACV compared to other host species, or that they are clearing infections and not seroconverting, although this would require further investigation. 

Considering the eastern gray squirrel (Order: Rodentia, Family: *Sciuridae*), a key reservoir host elsewhere, it has been reported that 39% (*n* = 140) of gray squirrels have neutralizing antibodies against LACV at a threshold of PRNT(50) [34]. Here, we detected a seroprevalence rate of 1.89% from the 264 gray squirrels sampled, which is much lower than that of other reported rates. This could illustrate reduced enzootic transmission in North–Northwestern VA or, similarly, indicate that the gray squirrel plays a lesser role in LACV transmission here. 

Experimentally, groundhogs (Order: Rodentia, Family: *Sciuridae*) have been found to maintain LACV-titers high enough to likely facilitate transmission to other mosquitoes, implicating them as potential reservoir species [35]. This has, however, only been confirmed in laboratory studies and not in wild groundhogs. Our current study did identify one naturally seropositive groundhog sample among the 56 groundhogs examined. Further screening of groundhogs could aid in determining a more precise seroprevalence rate; however, this is the first detection of antibodies against LACV in wild groundhogs.

Eastern cottontails (Order: Lagomorpha, Family: *Leporidae*) are not sciurid species more traditionally associated with LACV transmission, but have been identified with neutralizing antibodies against LACV (at rates of <1−15%) in Pennsylvania and Wisconsin [34,38]. The exposure identified in the cottontails tested in our study, 2.29% (3/131), is similar to LACV seroprevalence rates previously reported for cottontails. 

Red foxes (Order: Carnivora, Family *Canidae*) are known to seroconvert to LACV exposure, and in an experimental study gained infection when fed upon by a LACV-infected mosquito and facilitated onward transmission to naive mosquitoes that were able to then act as vectors, suggesting the role of red foxes as an amplifying host [35]. The current study identified neutralizing antibodies against LACV in one red fox (2.56% seroprevalence; *n* = 39). This is the first reported LACV seroprevalence rate designated to wild red foxes.

Over three years of sampling (2019 being excluded here due to collections starting late in the year, the lack of seropositive samples, and minimal sample size), LACV seroprevalence did increase. There was one seropositive individual detected in 2020 (*n* = 138, 0.7%), three detected in 2021 (*n* = 143; 2.1%), and six detected in 2022 (*n* = 231; 2.6%); although yearly variation was non-significant (Chi-Squared *p*-value = 0.58). Although these data do show an increase in seropositive samples, it is important to note that effort across the years (adjusted for here) varies due to the nature of passive sampling, and sample sizes per year are relatively low. The yearly increase may, however, indicate a rising risk of LACV exposure in Northern Virginia and require additional surveillance over future years to determine those risks. No significant difference was found between sex of animal, age, or county (Chi-Squared *p*-values = 0.17, 0.85, and 0.89, respectively).

Importantly, while serosurveillance indicates that a particular animal has been exposed, it does not indicate a current viremia or even recent exposure to LACV (except in the case of juvenile seropositivity). Therefore, it is possible that more mammals within the study had an active infection and had not yet seroconverted, that LACV neutralizing antibody titers have waned to non-detectable levels over time, or the possibility that maternal antibodies were passed onto offspring, although little research has been conducted in these topics. In humans, there is evidence that LACV neutralizing antibodies remain at high levels long after the onset of illness, suggesting that antibodies do not wane significantly, but data for non-human mammals are not available [39]. Although there is negligible information regarding maternal antibodies for LACV, there is a tentative suggestion of maternal antibodies providing protection for offspring against Jamestown Canyon virus (JCV), a related California serogroup virus; although, no definitive evidence nor duration were provided [30]. Maternal neutralizing antibodies against LACV may also be present in the six seropositive juvenile and sub-adult mammals that were screened here, which would reduce the exposure rates reported here. There is currently not an appropriate method for determining the difference in maternal antibodies and antibodies associated with a recent previous infection; however, maternal antibodies do still indicate that the mother was recently exposed to LACV and could be used as a proxy for LACV circulation. Additional research would be required to determine the role of maternal antibodies.

Seropositivity does not indicate where the LACV infection was acquired; however, since the animals listed here maintain relatively small ranges, except for red foxes, it is assumed to be local to the animal’s capture location. Eastern gray squirrels maintain home ranges of 2.4–3.4 hectares (ha) [40], eastern cottontails have a home range of 0.8–4.0 ha [41], and groundhogs have a home range of 0.39–1.99 ha [42]. Since these ranges are relatively small, compared to the size of VA, approximately 11-million ha [43], LACV-infections were likely acquired within the state and on a finer spatial scale, the county in which the animal was captured. Even with a larger home range, for example, red foxes are known to have a range of 237–558 ha [44], it is likely that infections were acquired locally to where the animal was captured.

VA has five health planning regions within the state that geographically coordinate with parts of the state.; Northern, Northwest, Southwest, Central, and Eastern Virginia [45]. Here, 313 samples (3 seropositive; 0.96% seropositivity) originated from the Northwest region (Albemarle, Augusta, Clarke, Culpeper, Fauquier, Fluvanna, Frederick, Orange, Page, Rappahannock, Rockingham, Shenandoah, Spotsylvania, Stafford, and Warren Counties and Charlottesville, Staunton, and Waynesboro Cities), 196 samples (7 positive; 3.57%) were from the Northern region (Alexandria, Arlington, Fairfax, Loudoun, and Prince William Counties), 8 (0 positives) originated from the Southwestern region (Augusta, Botetourt, Montgomery, and Roanoke Counties and Lynchburg City), 2 (0 positives) from the Eastern region (Northumberland County and Williamsburg City), and 1 (0 positive from the Central region (Richmond City). Seroprevalence rates vary across the state, with the largest seroprevalence rate in Northern and Northwest regions; other regions had no seropositive samples within the few samples tested from there. It is surprising that the Northern region had a higher seroprevalence than the Northwest region, since the Northwest is geographically located in proximity to the Appalachian region, which is known to have LACV cases (see further details below) [8,14,15,17]. Additionally, the Northern region is much smaller than other regions, yet comprises most seropositive samples. This may indicate the presence of a new LACV foci in the Northern part of the state.

Clinical cases of LACV in Virginia reported to ArboNET (CDC) between 2003 and 2019, originated from nine counties: Albemarle, Augusta, Dickenson, Fairfax, Henrico, Isle of Wight, Rockingham, Tazewell, and Wise counties [25]. Our data identified seropositive wildlife in Fairfax (*n* = 32; 3.13% seropositivity), but there was no other seropositive wildlife from the other counties with LACV clinical cases. Although there were wildlife samples from Albemarle (*n* = 5), Augusta (*n* = 3), and Rockingham (*n* = 3) counties, we were unable to evidence LACV circulation in these counties, likely due to the low number of wildlife samples available for screening.

Underreporting or under-detection in VA is concerning considering the severe disease that can result from LACV infection. It is possible that more people in Virginia are exposed to LACV but with few or mild symptoms; such infections are unlikely to be reported clinically. Serosurveillance in humans, for example, via screening blood-bank donations, is one method suggested to examine this further. 

Elsewhere, there is evidence that humans are exposed to many arboviruses including California serogroup viruses (of which LACV belongs). A study of park rangers, who were reported to be regularly exposed to mosquitoes that may carry arboviruses as an occupational hazard, showed a rate of neutralizing antibodies against any California serogroup virus of 30.9% [46]. Of four parks studied, the highest rates of LACV exposure originated from the Great Smoky Mountain State Park, a subrange of the Appalachians between NC and TN, with a seroprevalence ranging 22.7–24% [46,47]. This highlights the elevated exposure rate for those recreating or working outdoors and living in areas where mosquitoes are known to bite. It is possible that cross-reactivity to other California serogroup viruses occurs; JCV co-circulates with LACV in this region; however, previous studies have shown that LACV is minimally cross-reactive with JCV [48], and our conservative threshold for seropositivity makes the likelihood of cross-reaction low, and most seroposi-tive samples in our study showed a higher neutralization titer against LACV than JCV.

Overall, the seroprevalence in wild mammals highlights that LACV is circulating in parts of the Commonwealth of Virginia outside of the counties of Appalachia. Previous human cases in Northern VA, combined with the evidence of ongoing transmission of the virus, indicate the need for a more robust vector surveillance program to determine where LACV-exposure risks lie. General awareness of mosquito-borne viruses, such as LACV, and proper countermeasures may be a valuable tool in reducing future LACV clinical cases.

## 5. Conclusions

This study primarily indicates that LACV is circulating within non-Appalachian Virginia, but at lower rates than what is reported for endemic regions within VA and other states. Further research is needed to determine the extent that humans are exposed to LACV exposure via biting mosquitoes, and how that risk correlates to LACV circulation in wild mammal species. We identified a lack of LACV exposure in sciurid species, but we did find evidence of circulation and exposure in other mammal species. However, more research needs to be conducted to determine the role of these mammals in LACV transmission or potential reservoirs.

## Figures and Tables

**Table 1:** Wildlife species, number of individuals tested, and the LACV-seroprevalence rate for each species when positive at serotiter of 1:40 or greater.

Species	Number of Samples	LACV-Seroprevalence (when N > 15)
Eastern Gray Squirrel	264	1.89%
Eastern Cottontail	131	2.29%
Groundhog	56	1.79%
Red Fox	39	2.56%
Eastern Chipmunk	23	0%
Eastern Fox Squirrel	5	-
Flying Squirrel	5	-
American Beaver	3	-
Gray Fox	1	-
Total	527	1.90%

**Table 2:** LACV-seropositive animals detected in the study, including the date collected, county of origin, and corresponding PRNT(80) serum end-point titer. Sex is indicated as M (male), F (female), or U (unknown).

Species	Date Sampled (MM/DD/YYYY)	Age	Sex	VA County of Origin	Sero End-Point Titer
Eastern Gray Squirrel-1	06/06/2020	Juvenile	M	Frederick	1:160
Eastern Gray Squirrel-2	05/21/2021	Juvenile	U	Prince William	1:40
Groundhog-1	06/02/2021	Sub-Adult	U	Loudoun	1:320
Eastern Gray Squirrel-3	09/09/2021	Juvenile	F	Loudoun	1:320
Eastern Cottontail-1	03/31/2022	Infant	U	Warren	1:40
Red Fox-1	04/03/2022	Adult	U	Loudoun	1:40
Eastern Cottontail-2	04/29/2022	Adult	F	Loudoun	1:40
Eastern Gray Squirrel-4	07/17/2022	Adult	M	Fairfax	1:40
Eastern Cottontail-3	09/20/2022	Juvenile	U	Loudoun	1:40
Eastern Gray Squirrel-5	10/03/2022	Juvenile	F	Culpeper	1:80

## Data Availability

Data presented in this article are available upon request from the corresponding author.
